# Participation levels of physical activity programs for community-dwelling older adults: a systematic review

**DOI:** 10.1186/1471-2458-14-1301

**Published:** 2014-12-18

**Authors:** Marielle van der Deijl, Astrid Etman, Carlijn B M Kamphuis, Frank J van Lenthe

**Affiliations:** Department of Public Health Erasmus MC, P.O. Box 2040, 3000 CA Rotterdam, Netherlands

**Keywords:** Elderly, Participation level, Exercise, Intervention

## Abstract

**Background:**

Although many physical activity (PA) programs have been implemented and tested for effectiveness, high participation levels are needed in order to achieve public health impact. This study aimed to determine participation levels of PA programs aimed to improve PA among community-dwelling older adults.

**Methods:**

We searched five databases up until March 2013 (PubMed, PubMed publisher, Cochrane Library, EMBASE, and Web of Science) to identify English-written studies investigating the effect of PA programs on at least one component of PA (e.g. frequency, duration) among community-dwelling populations (i.e. not in a primary care setting and/or assisted living or nursing home) of persons aged 55 years and older. Proportions of participants starting and completing the PA programs (initial and sustained participation, respectively) were determined.

**Results:**

The search strategy yielded 11,994 records of which 16 studies were included reporting on 17 PA programs. The number of participants enrolled in the PA programs ranged between 24 and 582 persons. For 12 PA programs it was not possible to calculate initial participation because the number of older adults invited to participate was unknown due to convenience sampling. Of the five remaining programs, mean initial participation level was 9.2% (±5.7%). Mean sustained participation level of all 17 programs was 79.8% (±13.2%).

**Conclusions:**

Understanding how to optimize initial participation of older adults in PA programs deserves more attention in order to improve the population impact of PA programs for community-dwelling older adults.

**Electronic supplementary material:**

The online version of this article (doi:10.1186/1471-2458-14-1301) contains supplementary material, which is available to authorized users.

## Background

The worldwide population is ageing rapidly. Between 2000 and 2050, the world’s population over 60 years will double from about 11% to 22% [1], and healthcare costs will rise substantially [2]. Participating in regular physical activity (PA) is important for older adults, since it has positive effects on muscle strength, flexibility, balance, falls risk, and occurrence of chronic diseases [3], and may prevent or delay loss of independent living [4]. Preventive measures aimed at increasing PA levels should focus on those aged 55 years and older since they have been found to be at increased risk of adverse outcomes such as frailty and disability [5, 6].

High initial and sustained participation in PA programs is important for achieving public health impact [7]. However, although many PA programs have been implemented and tested for effectiveness [8], strikingly little is known about the participation levels of these programs [9, 10]. For example, low-intensity programs with a small effect and high participation rates may have a higher overall impact as compared to high-intensity programs with large effects and low participation rates [11–13]. As such, the identification of PA programs with high levels of participation is important for the development of future PA programs. Therefore, a systematic review was conducted to determine participation levels of PA programs aimed to improve PA among community-dwelling older adults aged 55 years and older. Furthermore, it was investigated what program characteristics and characteristics of participants distinct PA programs with higher participation levels from PA programs with lower participation levels.

## Methods

### Search strategy

Specified search strategies were developed for five bibliographic databases up until March 2013: PubMed, PubMed publisher, Cochrane Library, EMBASE, and Web of Science. The full electronic search strategy for Pubmed was:

((aged NOT (boy* OR girl* OR child*OR month* OR middle)) OR elder* OR senior* OR (old* AND (adult* OR people*))) AND (((communit* OR home) AND (living OR dwell* OR residen* OR based OR population*)) OR (residential* NOT (care OR home OR facilit*)) OR in home OR at home OR domestic*))) AND (exerci* OR sports OR physical OR activity OR activities OR walking OR swimming OR cycling OR strength OR endurance OR power OR pedometer OR accelerometer) AND (program* OR intervention* OR experiment* OR (group AND lesson*) OR government*) AND (effectiv* OR evaluat* OR outcome* OR benefit*)

The search strategies for the other databases can be found in the Additional file 1.

### Study selection

Studies were included when they were: 1) written in English; 2) conducted among community-dwelling populations (i.e. not in a primary care setting and/or assisted living or nursing home); 3) among persons aged 55 years and older; 4) described programs targeting at least one component of PA (e.g. walking group, exercise class); and 5) evaluating the effect of at least one component of PA (e.g. frequency, duration). Studies were excluded when these: exclusively targeted older adults with a specific medical condition (e.g. dementia, depression), focused on cost-effectiveness; and/or reported on study protocols only.

One reviewer (MvdD) performed the initial selection of titles and abstracts in the literature search. A second reviewer (AE) was consulted to screen a random sub-set, and in case of doubt to discuss until agreement was reached. All corresponding authors of included studies were contacted and reference lists of previously published systematic reviews were checked to make sure all relevant articles were captured. This extra search did not result in extra studies eligible for inclusion.

### Data extraction

A data extraction form was used to collect information on participation levels (dependent variable) and characteristics of participants and program characteristics (independent variables). Characteristics of participants included sex distribution (% females) and mean age of the participants. The program characteristics included: sampling method (probability sampling vs. convenience); method of recruitment; location (home-based vs. group-based); content (e.g. walking group); duration (months); number of contacts; supervision (yes vs. no); and (maximal) group size. Probability sampling is a method of sampling that utilizes some form of random selection, whereas convenience sampling is a technique where subjects are selected because of their convenient accessibility and proximity to the researcher (e.g. inviting through advertisements). One reviewer (MvdD) performed the data extraction and a second reviewer (AE) verified all extracted data. In case of doubt, data were discussed until agreement was reached.

### Participation levels

In order to calculate participation levels the following measures were used, numbers of persons that: 1) were invited to participate (i.e. available sample); 2) started the PA program; and 3) completed the PA program. By using these measures initial and sustained participation levels were calculated. Initial participation was defined as the number of participants that enrolled in the program divided by the number of persons invited to participate. Sustained participation was defined as the number of participants who completed the program divided by the number of participants that started the program [7].

### Risk of bias

Studies reporting significant effects of PA programs on PA outcomes are more likely to be published as compared to studies in which no significant results were found. However, it is unlikely that this publication bias would affect our results since we focused on participation level as the main outcome, and no differences in participation level are to be expected between effective and non-effective PA programs.

### Statistical analysis

Descriptive statistics (e.g. means, standard deviations, ranges) were used to summarize the results. Mean sustained participation level was calculated for all PA programs as well as for *effective* PA programs only. An *effective* PA program was defined as a program for which a significant effect on at least one PA outcome was reported. Pearson correlations were calculated in order to investigate the correlation between participation levels and: gender distribution of the participants; mean age of the participants; program duration; and group size.

## Results

### Literature search

**Figure 1:**
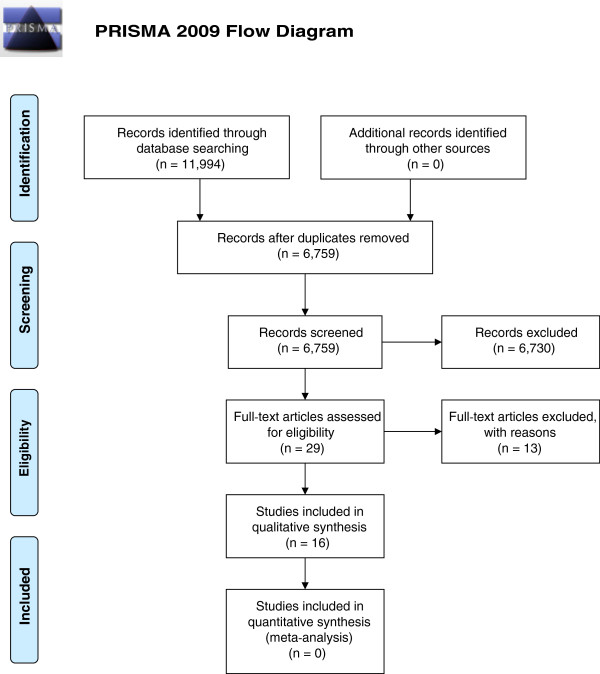
**PRISMA 2009 Flow Diagram.**

The search strategy yielded 11,994 records. After removing duplicates, 6,759 records remained which were screened based on title and abstract. Sixteen studies reporting on 17 PA programs, were included which were published between 2002 and 2013 since no studies prior to this time met the inclusion criteria (Figure 1).

### Characteristics of participants and programs

The mean age of the participants ranged between 66 to 84 years (overall mean 73.8 ± 6.6 years). In three programs only females participated [14–16]. Of the remaining 14 PA programs, on average 70.2% (±13.3%) of the participants were females (range 47-89%).

Program characteristics that showed the most variation were the location at which the program took place and the content of the program. Six programs were home-based [14, 15, 17–20], five programs were group-based [16, 21–24], and six were both home- and group-based [25–29]. Three programs involved group-walking [14, 16, 18], seven programs involved multifaceted activities such as a combination of education and a training program [15, 17, 20, 22, 25, 27], and seven programs involved various PA such as a pedometer intervention or different exercise programs [19, 21, 23, 24, 26, 28, 29] (Table 1). PA outcomes that were evaluated were: general PA level (n = 9); walking (n = 6); and household and sports activities (n = 1).

**Table 1:** **Characteristics of PA programs aimed at improving PA among community-dwelling persons aged 55 years and older (n=17)**

Participation level			Characteristics of participants		Program characteristics							
Study	Initial participation (%)	Sustained participation (%)	% Females	Mean age in years (range)	Sampling method	Method of recruitment	Home- or group-based	Content	Duration	Contacts	Supervision	Group size
Burke, 2013 [17]	16.2 (478/2,949)	78.4 (375/478)	48	66 (range not reported)	Probability sampling	From 60 suburbs/ neighborhoods of a city; random selection (method not reported)	Home-based	Multifaceted: booklet with PA and nutritional recommendations, resistance band, pedometer	6 months	6-10 telephone calls and/or 2-5 emails	No	0
Hopman-Rock^a^, 2002 [25]	12.9 (71/551)	70.4 (50/71)	63	Mean not reported (75-85)	Probability sampling	From a city selected and approached by mail for participation	Combination	Multifaceted; health education, group exercises, home exercises	3 months	Group meeting once plus exercises at home minimally 3 times/week	Yes	25
Cheng, 2009 [18]	8.2 (96/1,175)	79.2 (76/96)	47	70 (65-69)	Probability sampling	By using a list of names obtained from the department of households; letters were sent	Home-based	Walking training	3 months	3 meetings	Yes	0
Rosenberg, 2012 [26]	7.3 (87/1,196)	74.6 (64/87)	76	84 (69-98)	Probability sampling	From 4 senior-living facilities. Study information flyers were sent to all potential eligible residents	Combination	Standard intervention (pedometer, educational, group meeting) Enhanced intervention (+ telephone counseling, environmental awareness)	3 months	Biweekly group meeting	Yes	-
Rydwik, 2009 [27]	1.4 (96/6,999)	66.7 (64/96)	60	83 (range not reported)	Convenience sampling	By using questionnaires, advertisements in local newspaper, primary care and home service administration organized by the local authorities	Combination	Multifaceted: Training group (T) Group with training and nutrition (T+N) Nutrition-only group (N)	9 months	3 months twice a week training sessions (T and T+N), followed by 6 months home-based exercises (T)	Yes	5-8
Koizumi, 2009 [19]	-	100 (68/68)	100	67 (60-78)	Convenience sampling	From senior centers and advertisement in local papers	Home-based	Lifestyle PA: 9000 steps and 30 min moderate intensity PA per day	3 months	1 meeting individual	No	0
Shaw, 2008 [14]	-	100 (24/24)	100	Mean and range not reported	Convenience sampling	Four senior centers that host a program. One-on-one recruitment of other program participants and posting of signs in the center to announce the program	Home-based	A daily walking-for-exercise program at respective senior center (instruction to walk)	3 months	-	No	0
Bonnefoy, 2012 [15]												
	-	96.1 (98/102)	86	84 (78-97)	Probability sampling	Representatives and home helpers from associations involved in home assistance for the elderly, help to recruit people for participation	Home-based	Multifaceted; education meeting, nutrition supplement, 13 exercises per day at home	4 months	1 meeting	Yes	0
Fujita, 2003 [21]	-	95.4 (62/65)	54	67 (60-81)	Convenience sampling	From advertisements in local newspapers, bulletin, poster	Group-based	Progressive endurance training and resistance exercises	6 months	2-3 times/week	Yes	31
Sarkisian, 2007 [22]												
	-	90.2 (46/51)	89	77 (range not reported)	Probability sampling	From three senior centers (method not reported)	Group-based	Multifaceted; attribution retraining, PA class	1 month	Once a week	Yes	8-14
Croteau, 2007 [20]	-	82.6 (147/178)	78	73 (55-94)	Convenience sampling	From various educational, health, and social programs offered in area	Home-based	Multifaceted: pedometer, individual meeting, group meeting once a month	3 months	3 group- and 1 individual meeting	No	0
Opdenacker, 2008 [28]	-	78.3 (141/180)	49	67 (range not reported)	Convenience sampling	By using personal letters and advertisements on local radio and in local newspapers	Combination	Structural exercise intervention Home-based lifestyle intervention	11 months	3 times/week	Yes	10
Hernandes, 2012[23]	-	78.0 (238/305)	69	68 (64-71)	Probability sampling	Controls recruited among individuals participating in another project Experimental group recruited from 5 community-based exercise programs in the same city	Group-based	Community-based exercise program: aerobic exercises	4-40 months (on average 12 months)	2 times/week	Yes	13
De Vreede, 2007 [24]	-	75.5 (74/98)	100	74 (range not reported)	Convenience sampling	From advertisements in a local newspaper	Group-based	Functional tasks exercise program resistance strength exercise program	3 months	3 times/week	Yes	6-12
Michael, 2008 [16]	-	73.1 (424/582)	71	74 (range not reported)	Probability sampling	Neighborhoods in a city were selected demarcated by the city council	Group-based	Leader led walking group	6 months	3 times/week	Yes	10
Helbostad, 2004 [29]	-	68.8 (53/77)	81	81 (range not reported)	Convenience sampling	Invitations to participate were distributed by health care workers and by announcement in the local newspaper	Combination	Combined training: meeting at home, training classes	3 months	2 times/week	Yes	5-8
Hopman-Rock^b^, 2002 [25]	-	50.3 (196/390)	82	72 (55-75)	Convenience sampling	Advertisement in local newspapers and other media, personal communication, and a brochure	Combination	Multifaceted; health education, group exercises, home exercises	3 months	Group meeting once plus exercises at home minimally 3 times/week	Yes	22 (maximum 25)

### Initial and sustained participation

The number of participants enrolled in the PA programs ranged between 24 and 582, with a mean of 174 (±165). It was not possible to calculate initial participation levels for 12 PA programs, because their applied sampling methods (e.g. convenience sampling) made it unclear how many older adults were invited to participate. The mean initial participation level of the five remaining PA programs was 9.2% (±5.7%), with a range between 1% [27] and 16% [17]. It was not possible to calculate correlations of characteristics of participants and programs with initial participation levels because of the low number of studies reporting initial participation levels.

Between 24 and 424 (mean 129 ± 117) participants completed the PA programs. The mean proportion of persons completing the program was 79.8% (±13.2%; n =17) ranging between 50.3% [25] and 100% [14, 19]. Of the 12 *effective* PA programs [14, 16–20, 22, 25, 27–29] the mean proportion of persons completing the program was 71.3% (±21.9%). Correlations showed that higher sustained participation levels were related to lower mean age of the participants (r = −.182), higher proportions of females (r = .279), lower duration of the program (r = −.137), and smaller group sizes (r = −.367), but none of these correlations reached significance.

## Discussion

This systematic review identified 17 PA programs that aimed to improve PA among community-dwelling older adults. The mean proportion of participants starting the program (initial participation level) was 9.2%, but could only be calculated for five PA programs. The 17 PA programs had a mean sustained participation level of 79.8%. No significant correlations were found for participant or program characteristics with sustained participation level.

The mean initial participation level of 9.2% is difficult to interpret without additional information about the method of recruitment and effort or resources invested. For example 9.2% seems high when recruitment is done by putting up an advertisement in a community building, but low when mailing people personally and subsequently phoning them. Although for public health impact it is important to have insight into the number of older adults that would participate when providing a PA program [30], for 12 PA programs important information was missing. This is striking since information on initial participation gives insight into potential selective participation and in the external validity of the results. Furthermore, in the recent CONSORT statement it was emphasized to include information on the eligible participants in order to increase validity [31]. Thus, it is important that at least an indication of initial participation levels is reported when the effects of PA programs are studied. Therefore, for future studies it is highly recommended to include information regarding the number of persons that were invited to participate in the PA program. Although, none of the included PA programs in this current systematic review included online components, it is of interest to study the growing implementation of online PA programs [32] which potentially increase the ease of initial participation.

The overall mean sustained participation level of almost 80% found in the current systematic review was higher than expected, as lower participation levels have been found among children [33, 34], and for other types of health-behaviour programs for older adults [10]. The mean sustained participation level of *effective* PA programs was lower than the overall mean. This could imply that the *effective* programs have a smaller overall population impact when implemented on a larger scale as compared to programs with smaller effects but higher sustained participation levels [11–13].

No significant correlations were found for participant or program characteristics with sustained participation level which may be due to the small number of studies that were eligible for inclusion. Although the size of the correlations indicated that a low mean age of the participants, high proportions of females participating, short duration of the program, and a small group size are likely to increase levels of sustained participation, these factors should be investigated further as potential determinants of sustained participation. Jancey et al. (2007) showed that can be related to low socioeconomic status, overweight, low PA level at the start, low walking self-efficacy, and loneliness may also be related to low sustained participation levels of PA programs among older adults [35].

## Conclusions

Calculating initial participation levels of PA programs aimed to improve PA levels among community-dwelling older adults is hindered by high levels of convenience sampling. Sustained participation among those who started participating in PA programs is high. A low mean age of participants, high proportions of females participating, short duration of program, and a small group size are likely to increase levels of sustained participation. In order to improve the population impact of PA programs among community-dwelling older adults, more knowledge is needed into how initial and sustained participation levels can be optimized.

## Electronic supplementary material

Additional file 1: **Specified search strategies.** (DOCX 15 KB)
