# Supplementary material for: Participation levels of physical activity programs for community-dwelling older adults: a systematic review
Source: BMC Public Health. 2014 Dec 18;14:1301. doi: 10.1186/1471-2458-14-1301 (PMC4301079; doi:10.1186/1471-2458-14-1301)
Supplement: Supplementary file 1 — Additional file 1: Specified search strategies. (DOCX 15 KB) [file 12889_2014_7385_MOESM1_ESM.docx]

**Appendix**

PubMed publisher

(((((aged[tiab] NOT (((boy*[tiab] OR girl*[tiab] OR child*[tiab] OR month*[tiab] OR middle[tiab])))) OR elder*[tiab] OR senior*[tiab] OR (old*[tiab] AND (adult*[tiab] OR people*[tiab])))))) AND ((((communit*[tiab] OR home[tiab]) AND (living*[tiab] OR dwell*[tiab] OR residen*[tiab] OR based[tiab] OR population*[tiab])) OR (residential*[tiab] NOT (((care[tiab] OR home[tiab] OR facilit*[tiab])))) OR in home[tiab] OR at home[tiab] OR domestic*[tiab])) AND ((exerci*[tiab] OR sports[tiab] OR physical[tiab] OR activity[tiab] OR activities[tiab] OR walking[tiab] OR swimming[tiab] OR cycling[tiab] OR strength[tiab] OR endurance[tiab] OR power[tiab] OR pedometer[tiab] OR accelerometer[tiab])) AND ((((program*[tiab] OR intervention*[tiab] OR experiment*[tiab] OR (group[tiab] AND lesson*[tiab]) OR government*[tiab])) AND ((effectiv*[tiab] OR evaluat*[tiab] OR outcome*[tiab] OR benefit*[tiab])))) AND publisher[sb]

Cochrane Library

(((((aged NOT (((boy* OR girl* OR child* OR month* OR middle) NEAR/3 (aged)))) OR elder* OR senior* OR (old* NEAR/3 (adult* OR people*))):ab,ti))) AND ((((communit* OR home) NEAR/3 (dwell* OR residen* OR based OR population*)) OR ((living) NEAR/3 (home OR communit*)) OR (residential* NOT (((residential*) NEAR/3 (care OR home* OR facilit*)))) OR ((in OR at) NEXT/1 home) OR domestic*):ab,ti) AND ((exerci* OR sports OR physical OR activit* OR walking OR swimming OR cycling OR strength OR endurance OR power OR pedometer OR accelerometer):ab,ti) AND ((program* OR intervention* OR experiment* OR (group* NEAR/3 lesson*) OR government*):ab,ti) AND ((effectiv* OR evaluat* OR outcome* OR benefit*):ab,ti)

EMBASE

('elderly care'/exp OR ((aged NOT (((boy* OR girl* OR child* OR month* OR middle) NEAR/3 (aged)))) OR elder* OR senior* OR (old* NEAR/3 (adult* OR people*))):ab,ti) AND (community/de OR 'community care'/exp OR 'residential area'/de OR 'community assessment'/de OR (((communit* OR home) NEAR/3 (dwell* OR residen* OR based OR population*)) OR ((living) NEAR/3 (home OR communit*)) OR (residential* NOT (((residential*) NEAR/3 (care OR home* OR facility*)))) OR ((in OR at) NEXT/1 home) OR domestic*):ab,ti) AND ('physical activity'/exp OR sports/exp OR exercise/exp OR 'physical education'/de OR (exerci* OR physical OR activit* OR walking OR swimming OR cycling OR strength OR endurance OR power OR pedometer* OR accelerometer*):ab,ti) AND ('community program'/de OR 'program development'/de OR 'health program'/de OR 'education program'/de OR government/de OR (program* OR intervention* OR experiment* OR (group* NEAR/3 lesson*) OR government*):ab,ti) AND ('comparative effectiveness'/de OR evaluation/de OR 'course evaluation'/de OR 'evaluation research'/de OR 'outcome assessment'/de OR (effectiv* OR evaluat* OR outcome* OR benefit*):ab,ti)

Web of Science

TS=((((((aged NOT (((boy* OR girl* OR child* OR month* OR middle) NEAR/3 (aged)))) OR elder* OR senior* OR (old* NEAR/3 (adult* OR people*)))))) AND ((((communit* OR home) NEAR/3 (dwell* OR residen* OR based OR population*)) OR ((living) NEAR/3 (home OR communit*)) OR (residential* NOT (((residential*) NEAR/3 (care OR home* OR facilit*)))) OR ((in OR at) NEXT/1 home) OR domestic*)) AND ((exerci* OR sports OR physical OR activit* OR walking OR swimming OR cycling OR strength OR endurance OR power OR pedometer OR accelerometer)) AND ((program* OR intervention* OR experiment OR (group* NEAR/3 lesson*) OR government*)) AND ((effectiv* OR evaluat* OR outcome* OR benefit*)))
